# Embryonal Rhabdomyosarcoma with Posttherapy Cytodifferentiation and Aggressive Clinical Course

**DOI:** 10.1155/2021/1800854

**Published:** 2021-11-08

**Authors:** Maniraj Jeyaraju, Regina Ann Macatangay, Ashley Taylor-King Munchel, Teresa Anne York, Elizabeth A. Montgomery, Michael E. Kallen

**Affiliations:** ^1^University of Maryland School of Medicine, Baltimore, MD, USA; ^2^Division of Pediatric Hematology/Oncology, Department of Pediatrics, University of Maryland School of Medicine, Baltimore, MD, USA; ^3^Department of Pathology and Laboratory Medicine, University of Miami Miller School of Medicine, Miami, FL, USA; ^4^Department of Pathology, University of Maryland School of Medicine, Baltimore, MD, USA

## Abstract

Rhabdomyosarcoma is the most common soft tissue sarcoma in children and adolescents. Embryonal rhabdomyosarcoma (ERMS), its most common subtype, is a malignant soft tissue tumor with morphologic and immunophenotypic features of embryonic skeletal muscle. The histologic findings in ERMS typically include a range of differentiation in rhabdomyoblasts from primitive to terminally differentiated forms, and the latter become more prominent after chemotherapy-induced cytodifferentiation. Several reports have shown therapy-related cytodifferentiation to portend a good prognosis in ERMS. We discuss the case of a pediatric patient who presented with ERMS of the orbit. Although her tumor showed extensive posttreatment cytodifferentiation and several other good prognostic clinicopathologic factors, it pursued an aggressive course, resulting in early metastasis and death. This case represents an unusual course and may be instructive as to the clinicopathologic features impacting prognostication, and ultimately the biology, of this aggressive family of tumors.

## 1. Case Report

**Figure 1:**
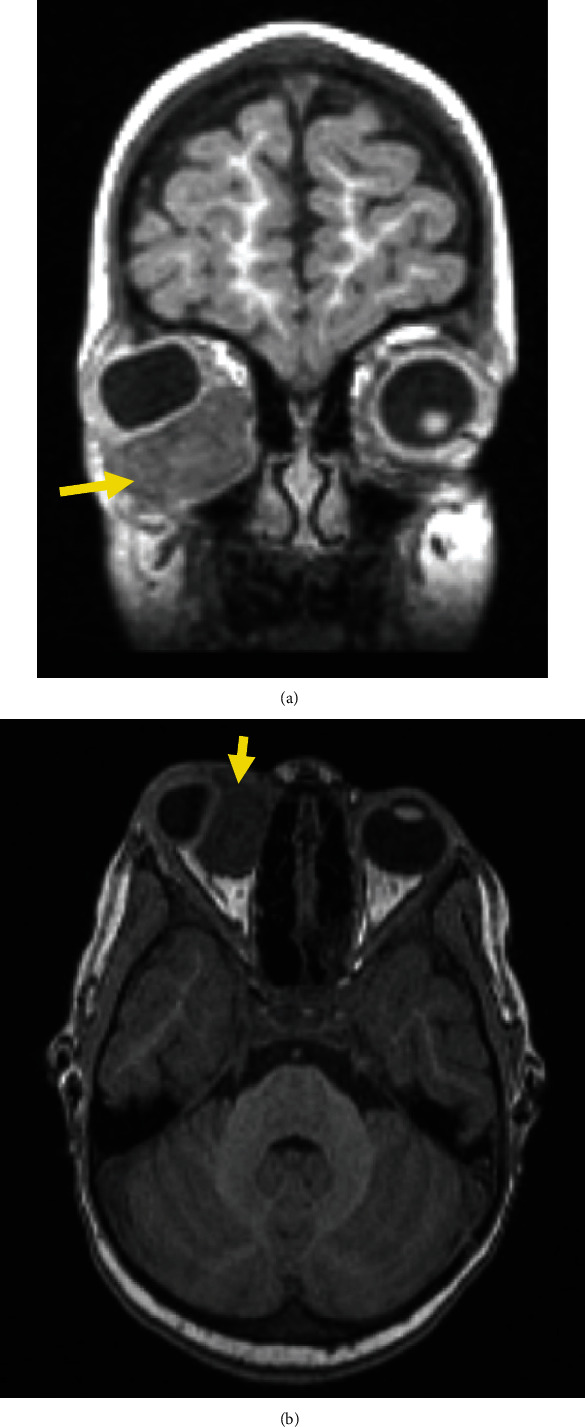
(a, b) MRI images demonstrate heterogeneously enhancing 2.7 cm extraconal mass lesion (yellow arrows), inferior to right globe and displacing it superolaterally.

**Figure 2:**
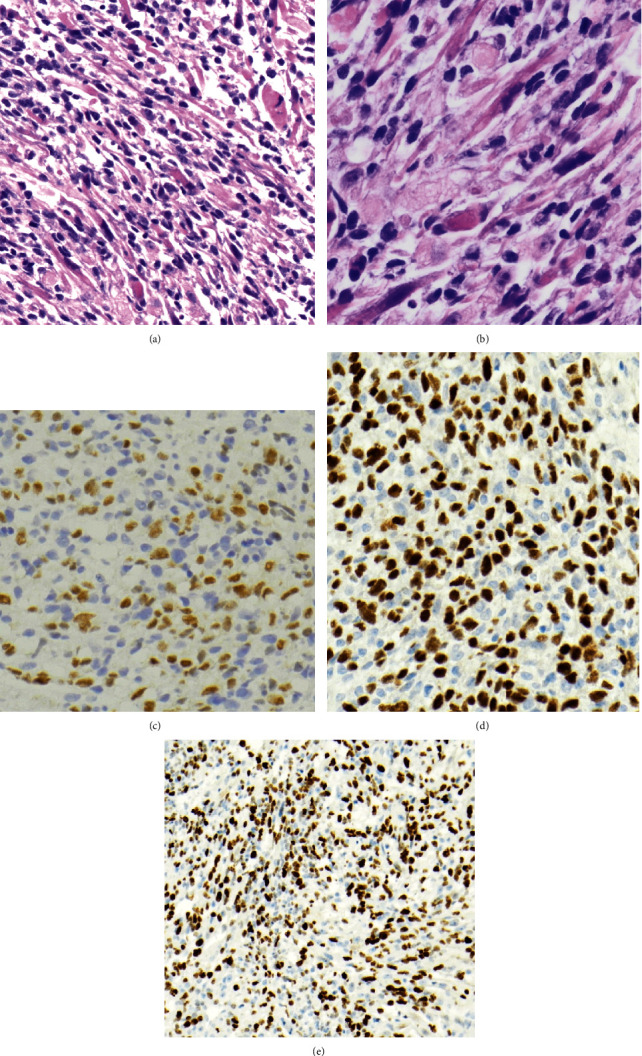
Histologic images from pretreatment incisional biopsy of the right orbital mass. H&E stains demonstrate a hypercellular proliferation of rhabdomyoblasts, showing a spectrum from primitive to differentiating forms, the latter of which feature elongated eosinophilic cytoplasm (strap cells) ((a) 100x magnification; (b) 400x magnification). Immunohistochemical stains demonstrate frequent myogenin staining of moderate intensity ((d) 100x magnification), strong myoD1 expression ((e) 100x magnification), and a high Ki67 proliferation index ((e) 40x magnification) in the rhabdomyoblasts.

A 4-year-old girl was brought to the pediatric emergency room with right eyelid swelling, tenderness, and pain with horizontal eye movement. Her past medical history included prematurity, developmental delay, febrile seizures, and an atrial septal defect. Magnetic resonance imaging (MRI) demonstrated a heterogeneously enhancing 2.7 × 1.8 × 3.0 cm mass, extraconal and isointense to the extraocular muscles, inferior to the right globe and displacing it superolaterally (Figure 1). An incisional biopsy demonstrated a hypercellular proliferation of primitive small round blue and spindled cells, featuring numerous strap cells and fascicular architecture (Figure 2). Immunohistochemistry demonstrated positivity for desmin, myogenin (patchy and moderate intensity), and MyoD1, as well as a high Ki67 proliferative index, confirming the diagnosis of ERMS. A p53 immunostain demonstrated frequent staining. Fluorescence in situ hybridization (FISH) testing was negative for *FOXO1* (*FKHR*) rearrangements. Further workup demonstrated no other sites of involvement, including negative marrow disease categorizing her as Stage 1 Group III orbital ERMS.

**Figure 3:**
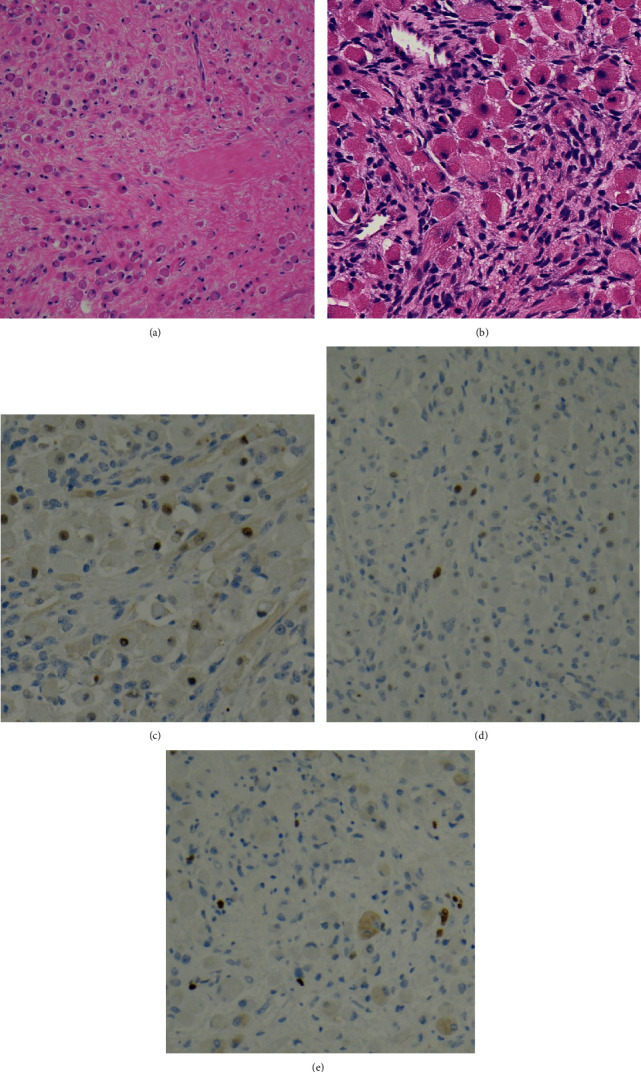
Histologic images from the posttreatment incisional biopsy of the right orbital mass. H&E stains demonstrate a variably cellular proliferation of terminally differentiated rhabdomyoblasts with eccentric nuclei and dense eosinophilic cytoplasm ((a) 100x magnification), with relatively few scattered foci showing primitive rhabdomyoblasts in the background ((b) 400x magnification). Immunohistochemical stains demonstrate scattered myogenin expression ((c) 100x magnification), infrequent myoD1 expression ((d) 100x magnification), and a low Ki67 proliferation index ((e) 100x magnification), all of which are markedly decreased in comparison to expression patterns from the pretreatment biopsy (Figure 2).

The patient was treated as per the Children's Oncology Group protocol ARST 1431 Arm C (not randomized) with vincristine, dactinomycin, and cyclophosphamide for 24 weeks, with radiation given after week 13. The end of therapy scans was stable and showed no active disease. While imaging initially showed slight improvement and reduction in size of the right orbital mass, surveillance MRI at 11 months after her initial presentation demonstrated a size increase to 3.1 × 1.5 × 3.2 cm. An incisional biopsy at this time point demonstrated sheets of mature rhabdoid cells (90%), in a sclerotic background, with only limited areas of primitive rhabdomyoblasts (10%) (Figure 3). Immunohistochemical stains showed strong desmin expression, and limited scattered myogenin and MyoD1 staining, with a variable and overall low Ki67 proliferation index, and very few p53 positive cells. The patient received salvage chemotherapy with vinorelbine, temsirolimus, and cyclophosphamide, but tumor size increased to 4.3 × 3.9 × 3.7 cm, prompting right maxillectomy and orbital exenteration; resection yielded negative margins and no lymph node involvement. She was treated postoperatively with 6 cycles of vincristine, cyclophosphamide, and topotecan; the family opted to stop therapy for increasing toxicity and no evidence of disease.

Unfortunately, 20 months after initial presentation, PET imaging demonstrated a 7.6 × 5.5 × 5.4 cm mediastinal mass with intense metabolic activity. A needle core biopsy of the mediastinal mass demonstrated sheets of primitive small round blue cells, numerous cells with prominent skeletal muscle differentiation, and frequent pleomorphic cells, with strong diffuse myogenin staining by immunohistochemistry, confirming the diagnosis of metastatic ERMS. Next-generation sequencing demonstrated mutations in *HRAS* (c.34G>A, variant allele frequency 66%), *PIK3CA* (c.1636C>G, variant allele frequency 35%), and *NF1* (c.7348C>T, variant allele frequency 32%).

She was enrolled to the Children's Oncology Group MATCH trial and randomized to a targeted treatment arm. Imaging demonstrated rapidly progressive thoracic disease that ultimately led to her death. Autopsy findings included a 20 cm mass in the left hemithorax, with left pleural involvement and encasement of the left lung, and numerous left lung and smaller right lung parenchymal metastases.

## 2. Discussion

Rhabdomyosarcoma (RMS) is the most common soft tissue sarcoma in children and adolescents, and embryonal is the most common subtype, with approximately half of cases occurring in the head and neck region including the orbit [1]. The histologic features of ERMS include mesenchymal cells in various stages of myogenesis, including primitive stellate cells, differentiating rhabdomyoblasts with elongation and eosinophilia (so-called tadpole or spider cells), and terminally differentiated cells with cross-striations or myotube formation [1]. ERMS typically demonstrates variably differentiated rhabdomyoblasts, in a loose myxoid background, with alternating areas of hypocellular myxoid and hypercellular spindled areas [1].

A notable finding in RMS after chemotherapy is that of cytodifferentiation, or proportional increase in rhabdomyoblasts further along the maturation spectrum, a finding that can be extensive, and occurs due to destruction of undifferentiated tumor cells [1–4]. Cytodifferentiation has been correlated with a favorable prognosis in ERMS and may identify patients whose tumors are more responsive to therapy, in data from several studies. In a series of 44 RMS cases of the bladder and prostate (39 embryonal or botryoid subtype, 5 alveolar subtype or other), Arndt et al. reported 4 recurrences in 17 patients with residual viable tumor, 2 recurrences and 1 toxic death in 17 patients with no viable tumor or mature rhabdomyoblasts, and 1 recurrence (and 1 patient lost to follow-up) in 10 patients with mature rhabdomyoblasts only [2]. Coffin et al.'s analysis of 16 RMS showed more frequent therapy-induced cytodifferentiation in ERMS and botryoid RMS (BRMS) than in alveolar RMS (ARMS) and an association with a favorable prognosis in BRMS. The relationship was less clear in ERMS and ARMS, where combined patterns of cytodifferentiation and residual undifferentiated foci were difficult to interpret [5]. Smith et al.'s study of 19 RMS showed 10 cases with >70% cytodifferentiation, in which all were BRMS or ERMS and none failed therapy, 5 cases with 30-70% cytodifferentiation in which only the ARMS cases failed therapy, 3 cases with <30% cytodifferentiation in which all failed therapy, and 1 case with no cytodifferentiation which failed therapy [6]. The finding of mature rhabdomyoblasts after chemotherapy, with a low mitotic index, has been suggested as an indication to cease adding additional treatment [3, 7].

The finding of well-differentiated rhabdomyoblasts after chemotherapy, in addition to portending a favorable prognosis in ERMS, also poses a potential diagnostic issue and complicates the interpretation of posttherapy biopsies for evaluation of residual disease. Arndt et al.'s report described 16 of 42 cases with conflicting pathology reports in posttreatment biopsies, both over- and underdiagnosing active tumor, and with variations in terminology including “differentiated,” “post-chemo,” and “treated” RMS [2]. The presence of a small number of undifferentiated tumor cells without treatment effect/differentiation have not been regarded as an indicator of an anticipated adverse outcome. Immunohistochemical stains for desmin, myogenin, and MyoD1 are useful in identification of myogenic precursors, though a pitfall is labeling in regenerating skeletal muscle.

Given the correlation between therapy-induced cytodifferentiation and a favorable prognosis in ERMS, our patient's unfortunate case represents an outlier, as she initially had extensive cytodifferentiation (though no marked shrinkage in tumor size preresection), followed by an aggressive clinical course with early metastasis and death. Notably, she also met several other favorable prognostic indicators in ERMS, including age (children ages 1-9 have improved prognosis over infants, adolescents, and adults), site (head and neck is a favorable site), and stage (Intergroup Rhabdomyosarcoma Study Group grouping system data showed Stage I). Compared to the initial biopsy, her posttreatment “cytodifferentiated” biopsy showed retained strong and diffuse desmin expression but slightly decreased myogenin and markedly decreased myoD1 positive cells, as well as a marked decrease in the Ki67 proliferative index. Decreased posttreatment expression of myogenin and MyoD1, transcriptional regulatory proteins expressed early in skeletal muscle differentiation, has been reported in a case of ERMS of the bladder, and is of uncertain significance [8]. A decreased posttreatment Ki67 proliferative index is also believed to suggest a favorable prognosis, although the immunostaining pattern can be difficult to interpret in the posttreatment context [6].

The explanation for our patient's aggressive clinical course may ultimately lie with the tumor's genetic features. Genomic studies of ERMS have identified a plethora of mutations involved in genetic syndromes and tumor development [9, 10], though the WHO particularly focuses on recurrent mutations involving the *RAS* pathway (*NRAS*, *KRAS*, *HRAS*, *NF1*, and *FGFR4*), effectors of PI3K (*PTEN*, *PIK3CA*), cell cycle controlling genes (*FBXW7*, *CTNNB1*), *NF1* mutations in 10% of cases, and others including *TP53* [1]. Approximately 30% of ERMS harbor multiple driver mutations; our patient was found to have mutations in 3 of these listed alterations (*HRAS*, *PIK3CA*, and *NF1*) by the next-generation sequencing assay. Additional mutations described in rhabdomyosarcoma include *BCOR*, *MEF2*, *MYOG*, *Ptch1*, *Gli1*, *Gli3*, *Myf5*, *MyoD1*, *IGF1R*, *PDGRFA*, *ERBB2/4*, and *MET* [9, 10], as well as *DICER1*, and gains in the *PAX3*, *DDIT3*, *Gli1*, and *Wnt* genes [11]. Larger cohorts will be required for a deeper understanding of the genetic landscape in ERMS and its clinical impact and will hopefully lead to improved targeted therapies in future patients.

## 3. Conclusion

Embryonal rhabdomyosarcoma often shows histologic features of cytodifferentiation in posttherapy specimens, a finding associated with an improved prognosis. We present the exceptional case of a pediatric patient whose tumor showed extensive cytodifferentiation and other favorable prognostic factors including embryonal subtype, orbital location, pediatric age group, and low stage. Regardless of these favorable factors, her tumor displayed a highly aggressive clinical course with distant metastasis and rapid demise. The specific biologic factors behind her case are poorly understood, though our expanding knowledge of the mutational landscape in sarcomas holds some promise for future targeted therapies. Until sufficient numbers of rhabdomyosarcoma cases are reported, it will remain difficult to correlate clinicopathologic features and prognosticate with certainty.

## Data Availability

The data used to support the findings of this study are included within the article.
